# Effects of Fineness and Chemical Composition of Blast Furnace Slag on Properties of Alkali-Activated Binder

**DOI:** 10.3390/ma12203447

**Published:** 2019-10-21

**Authors:** Abeer M. Humad, Karin Habermehl-Cwirzen, Andrzej Cwirzen

**Affiliations:** 1 Building Materials group, Structural and Fire Division, Department of Civil, Environmental and Natural Resources, Luleå University of Technology (LTU), 97187 Luleå, Swedenandrzej.cwirzen@ltu.se (A.C.); 2 Civil Engineering Department, University of Babylon, Babylon, Iraq

**Keywords:** alkali-activated slag GBFS, strength, microstructure of AAS, hydration products, shrinkage

## Abstract

The effects of fines and chemical composition of three types of ground granulated blast furnace slag (GGBFS) on various concrete properties were studied. Those studied were alkali activated by liquid sodium silicate (SS) and sodium carbonate (SC). Flowability, setting times, compressive strength, efflorescence, and carbonation resistance and shrinkage were tested. The chemical composition and microstructure of the solidified matrixes were studied by X-ray diffraction (XRD), thermogravimetric analysis (TGA) and scanning electron microscopy (SEM) coupled with EDX analyser. The results showed that the particle size distribution of the slags and the activator type had significantly stronger effects on all measured properties than their chemical composition. The highest compressive strength values were obtained for the finest slag, which having also the lowest MgO content. SC-activated mortar produced nearly the same compressive strength values independently of the used slag. The most intensive efflorescence and the lowest carbonation resistance developed on mortars based on slag containing 12% of MgO and the lowest fineness. The slag with the highest specific surface area and the lowest MgO content developed a homogenous microstructure, highest reaction temperature and lowest drying shrinkage. Thermogravimetric analysis indicated the presence of C-(A)-S-H, hydrotalcite HT, and carbonate like-phases in all studied mortars.

## 1. Introduction

Alkali-activated slag (AAS) binders are produced by alkaline activation of water-cooled ground granulated blast furnace slag (GGBFS), which is an industrial by-product from steel production. Commonly used alkaline activators include sodium hydroxide, sodium silicates, sodium carbonate and sodium sulphate in solid state to use it as one-part alkali-activated based binder or in liquid form [1].

AAS is considered as a sustainable alternative binder to Portland cement (PC) enabling a reduction in the CO_2_ emission of up to 80% compared to traditional concretes [2,3]. The solidified binder matrixes based on alkali-activation usually have a finer pore structure, lower water permeability as well as lower ion and molecular diffusivities in comparison with PC based systems [4,5]. Alkali-activated slag systems usually show a rapid strength development and often a rapid setting. The resistance to chemical attack is in most cases very high [6,7,8]. On the other hand, the AAS-based binder matrix has higher autogenous and drying shrinkage [9,10,11]. The drying shrinkage in PC systems tends to increase with decreasing relative humidity [12]. On the contrary, concretes based on AAS appeared to shrink more when exposed to a higher relative humidity. This was related to the reorganization of the C-A-S-H phase where alkali cations decrease the stacking regularity of the C-A-S-H layers and cause their collapse during drying [13]. The mineralogical composition, the fineness of the slag and the type and dosage of the alkaline activator were indicated as the most critical factors controlling the dissolution rate and solidification processes [14,15]. The main hydration products of AAS systems are calcium silicate hydrates incorporating aluminium C-(A)-S-H, hydrotalcite with Mg/Al ratios between 2 and 3, AFm and strätlingite [15,16]. The presence of small amounts of crystalline phases and a higher MgO content, increased the compressive strength, whereas more of Al_2_O_3_ resulted in its decrease [17].

The often observed efflorescence of alkali-activated binder systems is influenced by the pore structure and properties of the precursor [18].

The increased MgO content in the GGBFS enhanced the soundness of the autoclaved Portland cement-slag blends when the MgO was present in non-reactive phases including for example merwinite. On the other hand, the presence of MgO in more reactive phases has shown negative effects [19]. A higher MgO content of sodium silicate-activated slags resulted in a rapid strength development, higher ultimate compressive strength, lower porosity, and higher hydration heat [17,20]. Addition of a highly reactive MgO produced by calcination of MgCO_3_ at temperatures below 1000 °C to sodium silicate-activated-slag paste accelerated the early stage reaction, reduced the drying shrinkage and increased the carbonation resistance [21,22]. Those effects were related to the formation of C-M-S-H, having more polymerized units, stronger bond strength and better carbonation resistance in comparison with the C-S-H [23]. On the other hand, it also generated a severe cracking under drying condition [21,22]. The incorporation of MgO into AAS appeared to reduce the Al substitution in the C-S-H and resulted in a formation of more hydrotalcite phases [20].

On the contrary, the dead-burned MgO naturally present in GGBFS lowered and formed at 1500–1600 °C lowered the hydration degree [24]. Brucite (Mg(OH)_2_) was not detected in MgO-alkali-activated slag system, therefore suggesting the interaction between MgO and broken Si-O and Al-O, which possibly forms magnesium silicate hydrate (MSH) and expansive hydrotalcite-like phase (HT), which decrease the drying shrinkage strains of both alkali-activated slag and OPC [14].

Carbonation of C-S-H or (C-A-S-H) is essentially a chemical degradation process controlling the strength and durability of both AAS and OPC binders. The transformation of C-(A)-S-H into an amorphous silica gel and leaching of Ca from C-(A)-S-H results in a formation of an amorphous calcium carbonate (ACC) before its transformation into a more stable CaCO_3_ polymorph [24]. The MgO content in slag was observed to control the rate and the degree of the carbonation of AAS systems [14]. A higher MgO content lead to the formation of a stable ACC in AAS and increased the amount of formed hydrotalcite-like phase Mg_6_Al_2_CO_3_(OH)_16_·4(H_2_O) [25]. The trend was especially pronounced in sodium hydroxide-activated systems. The Al uptake by the C-S-H was decreased resulting in a reduced carbonation of the C-(A)-S-H [20,22,26]. Formation of a stable amorphous calcium carbonate lowered the solubility of the high MgO content slag by creating an alternative buffering system. The system reduced the decalcification, dehydration, and polymerization of C-A-S-H under accelerated carbonation conditions. The formed hydrotalcite-like phase would effectively bind CO_2_ and therefore hinder later carbonation [22]. Low-MgO AAS pastes formed amorphous calcium carbonate which crystallized quickly into calcite/vaterite, which is followed by further decalcification of the C-A-S-H gel [26].

The reaction heat development of the SS activated BFS systems was affected by the sodium content and the alkali modulus (M_s_) [27]. Higher Na_2_O content led to a more intensive breakage of the slag oxides bonds Ca-O, Mg-O, Si-O-Si, Al-O-Al, and Al-O-Si, which was correlated with the visible initial peaks. The second temperature rise occurred due to the formation of the Si-Al layers on the surface of the slag particles. In the final step, the reaction products formed through a condensation process. The MgO content of the slag seemed to effect the reaction kinetics especially with low concentrations of the alkali activator, while, the CaO content was more critical at higher concentrations [28].

Summarising, the reaction kinetics appeared to be affected by the chemical composition and physical properties of the slag precursors as well as the used type and dose of the alkali activator [28,29]. However, the number of studies focusing on special slags containing for example higher MgO content is rather limited. This study fills this gap by determining the effects of chemical composition of slag, MgO content, and other basic raw materials properties on strength, microstructure, and chemical composition of alkali activated mortars based on GGBFS.

## 2. Materials and Methods

**Figure 1:**
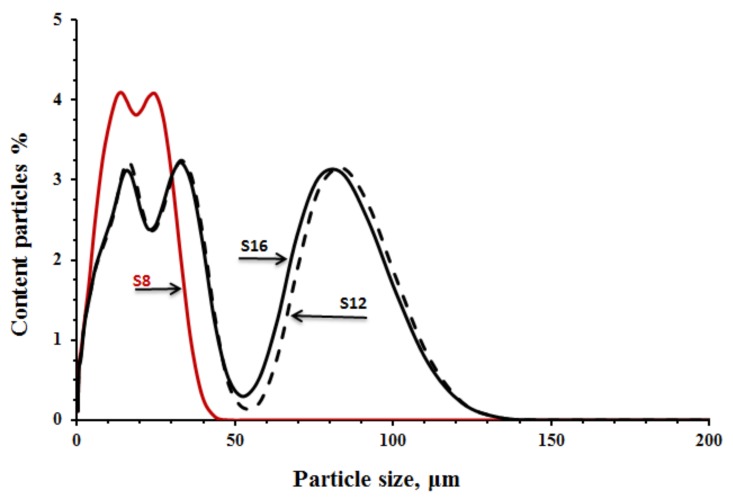
Particles size distribution of slags.

**Figure 2:**
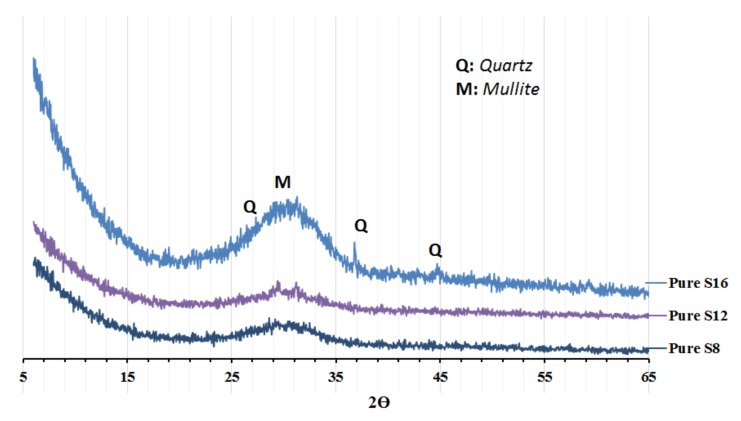
XRD analysis of the slags.

Three ground granulated blast furnace slags GGBSs with different MgO contents of 16.1 wt % (S**_16_**), 12.1 wt % (S**_12_**), and 7.78 wt % (S**_8_**) were used. The S**_16_** and S**_12_** slags were Merit 5000 provided from Merox AB-Sweden, while the S**_8_** slag was provided from Thomas CEMENT/Bremen, Germany. Chemical composition, physical properties, basicity (K_b_), and hydration modulus (HM) are shown in Table 1. The particle size distribution, shown in Figure 1, was determined with an LS 13 320 XR laser Particle Size Analyser (tested in Espoo, Finland). The XRD spectra of the untreated GGBFS are shown in Figure 2.

The basicity modulus (K_b_) and hydration modulus (HM) values were calculated for the three slags using the following equations; K_b_ = (CaO + MgO)/(SiO_2_ + Al_2_O_3_) and HM = (CaO + MgO + Al_2_O_3_)/SiO_2_).

Two alkaline activators were used: liquid sodium silicate (SS) Na_2_SiO_3_ provided by PQ Corporation (Karlstad, Sweden) and powder sodium carbonate (SC) Na_2_CO_3_ provided by CEICH SA (Warsaw, Poland). The supplied SS had an alkali modulus calculated as M_s_ = SiO_2_/Na_2_O (mass ratio) of 2.2, with 34.37 wt % SiO_2_, 15.6 wt % Na_2_O, and a solid content of 49.97 wt %. The M_s_ value was adjusted to 1.0 by adding sodium hydroxide pellets (98% purity), with 76.31 wt % Na_2_O. The alkali activator dosage was 10 wt % as a solid content of the binder weight for all mixes including pastes and mortars.

The water content of the water glass was taken into account in the mix design. The activators were dissolved in the mixing water the day before preparation of the mixes. The w/b ratio was 0.63 for pastes and 0.45 for mortars. Pastes and mortars for the determination of the setting time, efflorescence, carbonation, and heat development were prepared using a Hobart mixer. The dry materials were mixed for one minute, followed by the addition of the alkali activators dissolved in water and mixed for another 2 min. Mortars and pastes used to determine the mechanical properties were prepared in a small volume vacuum mixer type Ecovac-produced by Bredent/Senden, Germany. The mixing time was 2 min. The mortar had a w/b ratio of 0.45, with a binder/sand ratio of 1/1, Table 2.

**Figure 3:**
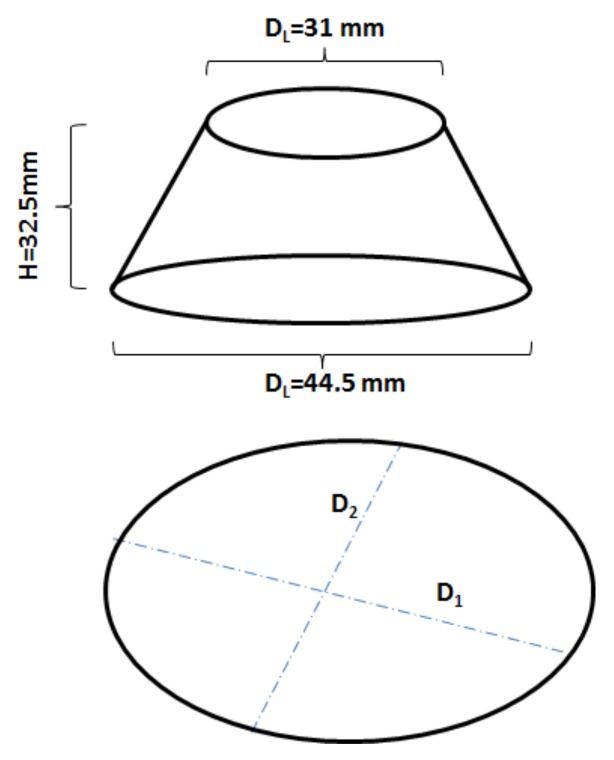
Schematic of the slump cone and spread flow diameter.

Initial and final setting times were determined using the Vicat apparatus on pastes (Form+Test Seidner&Co. Gmbh, Germany, tested in Sweden) following the ASTM C191-13 standard. A mini cone with dimensions of D_U_ = 31.0 mm, D_L_ = 44.5 mm and H = 32.5 mm was used to determine the workability of the produced mixes, Figure 3. The slump flow diameter D was determined as an average of the two measured values D_1_ and D_2_.

The temperature development was measured and recorded by using a thermocouple apparatus connected with the TC-08 produced by Pico technology (test in Sweden) using the PicoLog6 software (PicoSDK 10.6.13). The measurements were performed on paste cubes having dimensions of 60 × 60 × 60 mm^3^. The specimens were casted into isolated boxes, which were built of three layers of 20 mm thick polystyrene sheets. The temperature was recorded every minute for 156 hours at room conditions at 21 ± 2 °C and 35 ± 5% RH.

The compressive strength values were determined using mortar beams 12 × 12 × 60 mm^3^ tested at 7 and 28 d. After casting, the beams were sealed in plastic bags and stored at 21 ± 2 °C and 35 ± 5% RH until solidification. In the next step, samples were de-moulded and kept sealed in the same conditions until testing. The compressive strength was determined using a WTKEHAM FARRANCE compression device (made in England, tested in Sweden) using Catman Easy software (version 5.2.1) to measure the strength values. The loading speed was 0.05 mm/min.

Efflorescence and carbonation depth were determined using mortar prisms having dimensions of 40 × 40 × 160 mm^3^. All samples were casted and sealed in plastic bags for four days followed by their storage in unsealed conditions at 20 ± 2 °C and 50 ± 10% RH. The visual examination of efflorescence was done two days after opening of the moulds. The carbonation depth was determined three months later using a Phenolphthalein Deep Purple Indicator (GI, Copenhagen, Denmark) sprayed onto the split concrete surfaces.

The microstructure and microchemistry of the hardened AAS mortar samples were studied using a scanning electron microscope (SEM) model JSM-IT100 combined with a QUANTAX EDX (Energy-dispersive X-ray spectrometer) produced by BRUKER (made in Japan, tested in Sweden) and the ESPRIT 2 software (version 2.1). Samples used for the SEM and the SEM-EDX analyses were stored in isopropyl alcohol for 48 h to stop ongoing reactions. In the next step, the samples were impregnated with a low viscosity epoxy resin. No heat drying was applied to any of the studied samples to limit a possibility for artificial cracking of the matrix. After curing for 24 h, the resin-impregnated samples were grinded and polished in steps using polishing spray containing 9, 3, and 1 μm synthetic diamond particles. A load of 35 N was applied to the polished samples. The SEM was operating with a 15.0 kV accelerating voltage and a probe current of 60–62 mA. Backscattered electron images (BSE) (model JSM-IT100, tested in Sweden) in low vacuum mode were obtained.

The XRD analysis was done on powdered paste samples at the age of 28 d using a Panalytical Empyrean XRD (UK)unit with Cu Kα radiation, step size 0.0262° 2θ, the total scanning time for each sample was 16 min. The results were evaluated using the HighScore Plus software (v.4.0-4.7a).

The thermogravimetric analysis (TGA/DSC) (tested in Saraburi, Thailand) was done in a nitrogen gas atmosphere using a NETZSCH STA 409PC/PG TGA/DSC apparatus. The temperature range was set between 30 and 1000 °C, at heat rate of 10 °C/min was applied. Before the measurement, 28-day old samples were immersed in isopropyl alcohol for 48 h to stop ongoing reactions followed by grinding.

For the drying shrinkage test, two concrete cylinders the both having diameters of 100 mm and height of 200 mm were casted using 10 wt % SS added as solid materials. The binder content was 450 kg/m^3^, the w/b ratio 0.45, the maximum aggregate size was 8 mm and the fine aggregate content was 80% of the total aggregate amount, which was 1663 kg/m^3^. The specimens were sealed for three days, except for the S**_12_** mix, and stored in laboratory conditions at 20 ± 2 °C and 50 ± 10%, then kept unsealed in laboratory conditions. Three pairs of stainless steel studs were glued with an epoxy resin to the specimen surfaces. Strain values were recorded using an electronic manual strain gauge DEMEC type. A reference concrete mix was produced using rapid hardened PC cement with fineness of 520 m^2^/kg and w/c ratio of 0.45 with 1% plasticizer.

## 3. Results and Discussion

**Figure 4:**
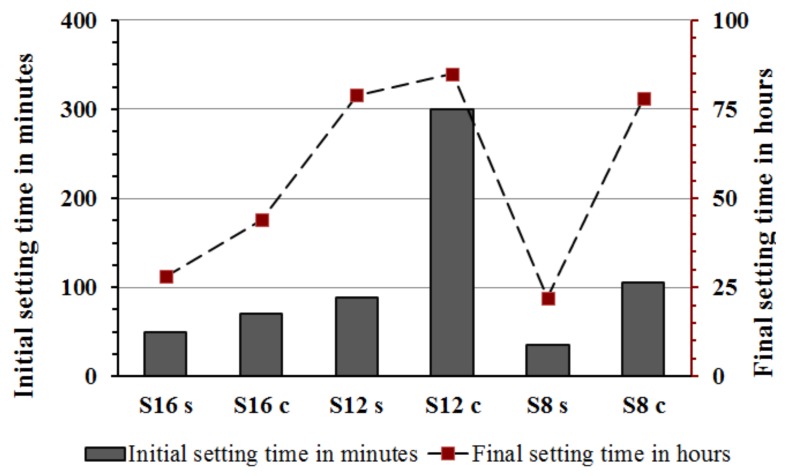
Initial and final setting time results of the AAS pastes.

**Figure 5:**
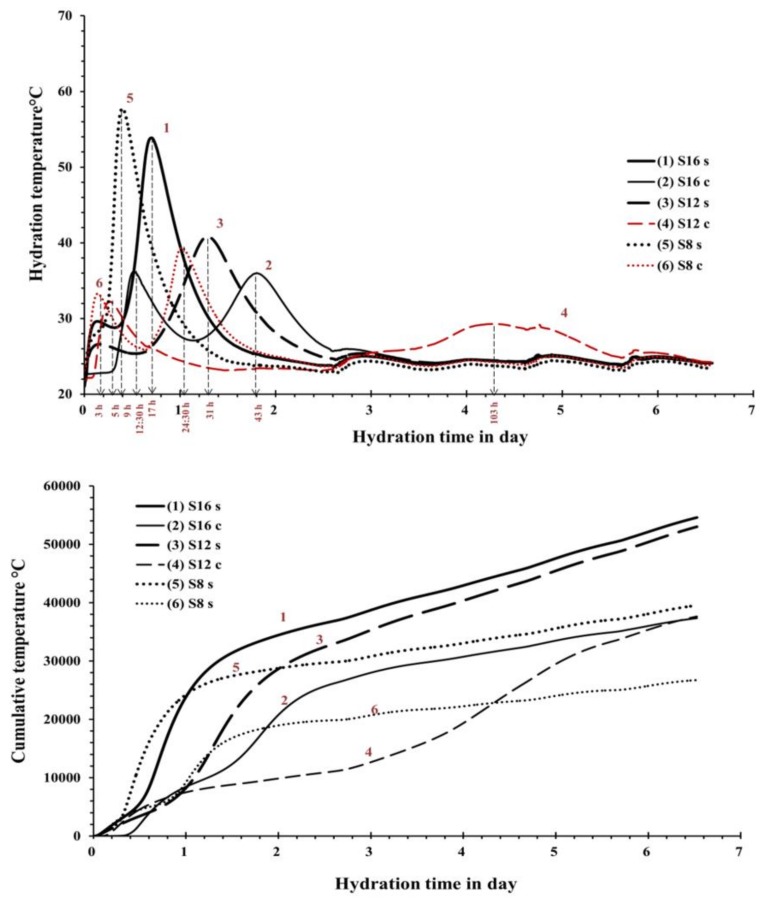
The hydration temperature peaks and the cumulative temperature of the AAS pastes.

The measured setting times were affected by fineness and chemical composition of the used slag and alkali activator Figure 4. The pastes activated with the SS showed generally shorter initial and final setting times in comparison with pastes activated with the SC. This is based on the higher pH values that led to a faster dissolution rate and increased reaction rate. On the contrary, the longest initial and final setting times were recorded for mixes activated with SC. The ultimate time length was strongly related to the fineness of the used GGBFS, which appeared to be the critical factor. The mix S**_12C_** contained the coarsest GGBFS and was activated with the SC showed an initial setting time of 300 min. This result can be directly related to a lower developed reaction temperature and thus slower reaction as observed also in the performed semi adiabatic measurement, Figure 5. Mixes with the longest initial setting times S**_12_s** and S**_12_c** showed also significantly lower maximum temperature as well as an elongated and delayed induction period. In addition to the coarsest particle sizes of this GGBFS, also the Al_2_O_3_ content was the lowest of all three tested slags Table 1. Earlier studies indicated that both the MgO and the Al_2_O_3_ content tend to affect the reaction speed of the SS-activated slag [20,30]. Those results showed that increasing the MgO content increased the reaction heat and the amount of the formed hydrotalcite but decreased the Al uptake by the C-S-H. The present results indicate the same trend. For example, when comparing setting times of mixes S**_16S_** and S**_12S_** the higher MgO content accelerated the setting. Both slags had nearly the same particle size distribution, Table 1 and Figure 1. The mixes made with the S**_8_** slag, which has the lowest content of MgO, the highest SiO_2_, CaO content and the highest specific surface area, showed the shortest setting times. The recorded temperature development was also the highest, independently of the used alkali activator Figure 5 (curve 5 and 6).

**Figure 6:**
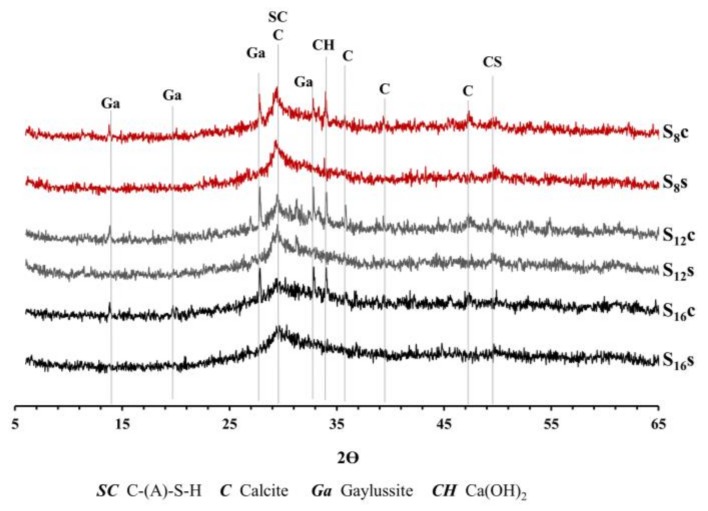
XRD results of the AAS pastes after 28 d of sealed curing at 21 ± 2 °C and 35 ± 5% RH.

The initial as well as the final setting times and the hydration temperature were comparable between mixes S**_8s_** and S**_16s_** activated with SS, Figure 4 and Figure 5. The same mixes but activated with SC showed longer initial setting times for mix using a finer slag S8. This could be related to a low pH of the SC solution (<12), which lead to a slow dissolution of the slag. Moreover, the Ca^2+^ released from the dissolved slag could react with the CO_3_^2−^ released from the activator (SC) to form carbonate salts like calcite and gaylussite, see XRD test results, Figure 6. This reaction takes place before the precipitation of C-(A)-S-H gel, consuming the Ca^2+^ released by the slag, and the later step of the reaction mechanism is similar to that of a NaOH-activated slag [31].

The XRD analysis showed the presence of calcite, gaylussite, as well as a larger amount of portlandite to be present in the SC-activated mixes, Figure 6. The recorded final setting times followed rather closely the same trends observed for the initial setting, Figure 5. The longest times were observed for mixes activated with SC and based on slags with the lowest Al_2_O_3_ content. Earlier studies also indicated an elongation of the final setting times for SC-activated slag pastes when compared with SS- or hydroxide-activated slag systems [7,32,33].

The semi-adiabatic calorimetry test results showed a significant discrepancy between mixes activated with SS and SC. The SS activated mixes showed only one main peak 9, 17, and 31 h after mixing for the mixes S**_8s_**, S**_16s_**, and S**_12s_** respectively. The location and the height of the peak can be directly related with the fineness of the slags, Table 1 and Figure 1. 

An earlier and more extensive heat development corresponded to higher fineness of the slag. Others indicated also that higher Na_2_O concentration could also increase the amount of the developed heat but it cannot be confirmed in the present studies [23,34]. An interesting difference was observed before the development of the main peak. Mixes S**_16s_** and S**_12s_** had similar pattern during the first 40 h with a smaller peak forming indicating a following slight temperature drop. On the contrary, the mix S**_8c_** did no develop any early age temperature rise. The difference could be related to the MgO content, which was significantly higher for mixes S**_16s_** and S**_12s_.** A higher MgO content, as described earlier, tends to facilitate the formation of hydrotalcite, which could be visible as those early age temperature increase [22,30].

Mixes activated with the SC developed two main temperature peaks. The first peak occurred after 3, 5 and 12.5 h for the mixes S**_8c_**, S**_12c_**, and S**_16c_** respectively. The second peak developed after 24, 103 and 43 h for mixes and S**_8C_**, S**_12C_**, and S**_16_**c, respectively. The second peak appeared to be larger in all cases indicating a predominant precipitation of carbonate salts including calcite and gaylussite in the first hours of the reaction. The following increase of the pH through the release of hydroxide ions resulted in the formation of C-S-H-like phases [35].

The observed late formation of a wide peak in the case of the Mix S**_12C_** could be related to a lower fineness and a lower MgO content. Similar results were obtained earlier by others [35]. In that case, long induction periods were detected in SC-activated slag pastes and were strongly related to the chemical composition of the used slags. The second peak of the mix S**_8c_** formed earlier and was the highest indicating a rapid temperature development and intensive chemical reactions. The cumulative developed temperature measured for up to 6 d were significantly higher for the SS-activated slag pastes S**_16S_**, S**_12S_**, and S**_8S_**. The highest total heat released during the hardening process appeared to develop in the case of the mix S**_16s_** based on slag with the highest MgO% content. Enhanced hydration kinetics during early ages related to the formation of calcite and gaylussite [20].

**Figure 7:**
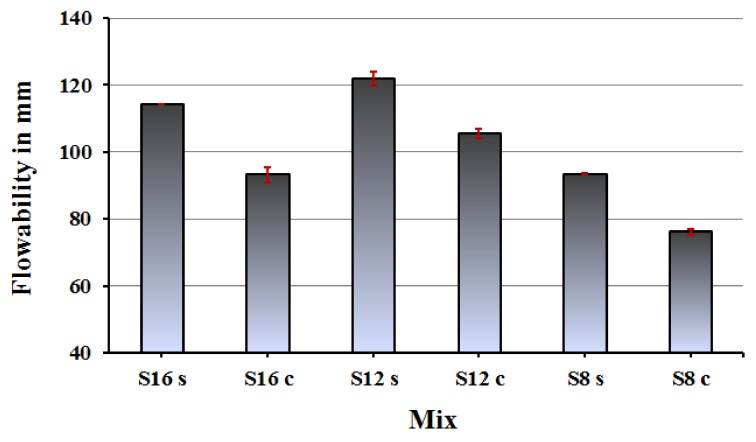
Flowability results of the AAS mortars as an average of the spread diameters.

The workability of fresh mixes was generally better when water glass was used as alkali activator, which is related to a lubrication effect Figure 7. Finer slags tended to lower the flowability as expected due to increase the partials surface area.

**Figure 8:**
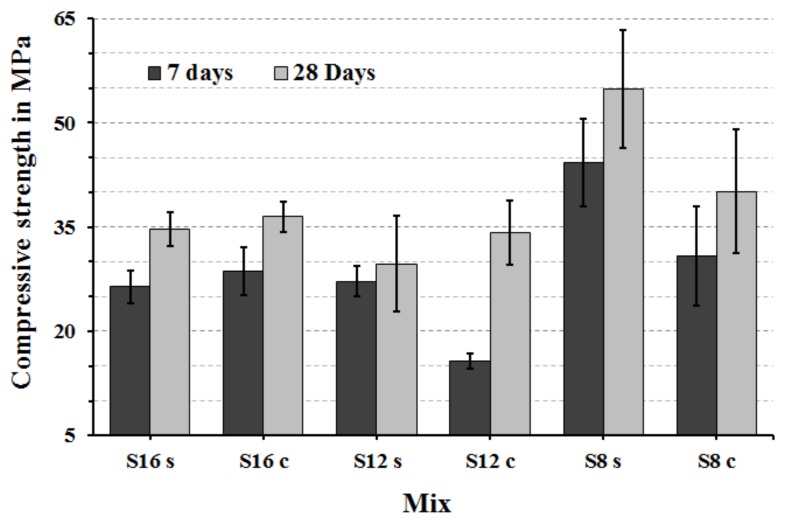
Seven and 28 d compressive strength results of the AASs mortars.

The highest measured 7 and 28 d compressive strength values were achieved by mixes based on fine S8 slag activated with sodium silicate, Figure 8. which complied with earlier results [7]. The lowest 7-day compressive strength values of the mix S**_12C_** are related to the slow hydration process and a long induction period Figure 5. At later stages that mix showed a rapid strength development and reached eventually nearly 35 MPa after 28 days. The highest compressive strength at both measured ages 7 and 28 d are recorded for mixes using fine slag S**_8_** and reached 44 and 55 MPa, respectively. The high strength values can be also related to higher contents of SiO_2_, CaO and MgO. The MgO/Al_2_O_3_ ratio for this particular slag was also the lowest of all used material, Table 1. The high recorded compressive strength is in agreement with a previous study, which tested mixes containing 30 wt % of Portland cement blended with different proportion of GGBFS based mortars. Those results indicated that mixes with the lowest MgO/Al_2_O_3_ ratio and high Al_2_O_3_ produced matrixes having the highest compressive strength but also a high amount of ettringite and some hydrotalcite [17].

The efflorescence is caused by migration of alkalis (Na^+^) in the pore solution to the surface, their reaction with atmospheric CO_2_ producing the Na_2_CO_3_. The extent of efflorescence in this study was determined by visual observation 2 d after removal of the seal. The used specimens were initially kept in sealed-moulds for four days, then opened and kept in the ambient conditions at 20 ± 2 °C and 50 ± 10% RH. The AAS mortar mixes based on the S**_12_** slag and activated with both SS and SC (Mixes S**_12S_** and S**_12C_**) showed the most severe efflorescence. The observed trend can be related to the slow reaction rate of mixes containing the S**_12_** GGBFS, Figure 5. The development of more porous microstructure especially at early age facilitated the migration of alkali to the surface. The efflorescence as such is structurally harmless [36]. Furthermore, the solubility of Na^+^ and leaching are limited, but the high mobility is due to the high dose of activator or the inherent neutralization of Al(OH)_4_ in pore solution, or both [18]. The EDX analysis of the observed efflorescence salts indicated only the presence of sodium carbonate. On the contrary, the efflorescence products in the OPC system is composed mainly of CaCO_3_ [36]. The Na-O bond is weakly connected with water molecules in the pore solution, on the contrary to the Na-O bond in the N-A-S-H [18]. The intensive micro-crack formation was observed in the case of S_16S_ and S_8C_.

**Figure 9:**
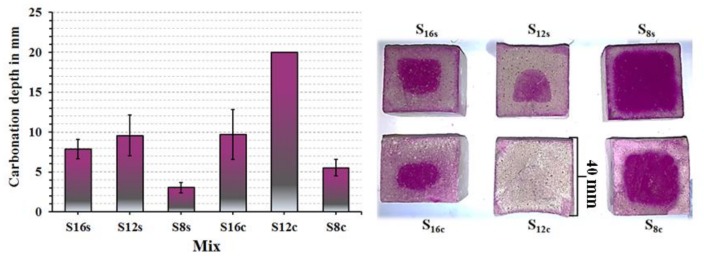
Carbonation depth of the AASs mortars specimens (40 × 40 mm^2^) determined after 3 months of storing at 20 ± 2°C and 50 ± 10% RH.

**Figure 10:**
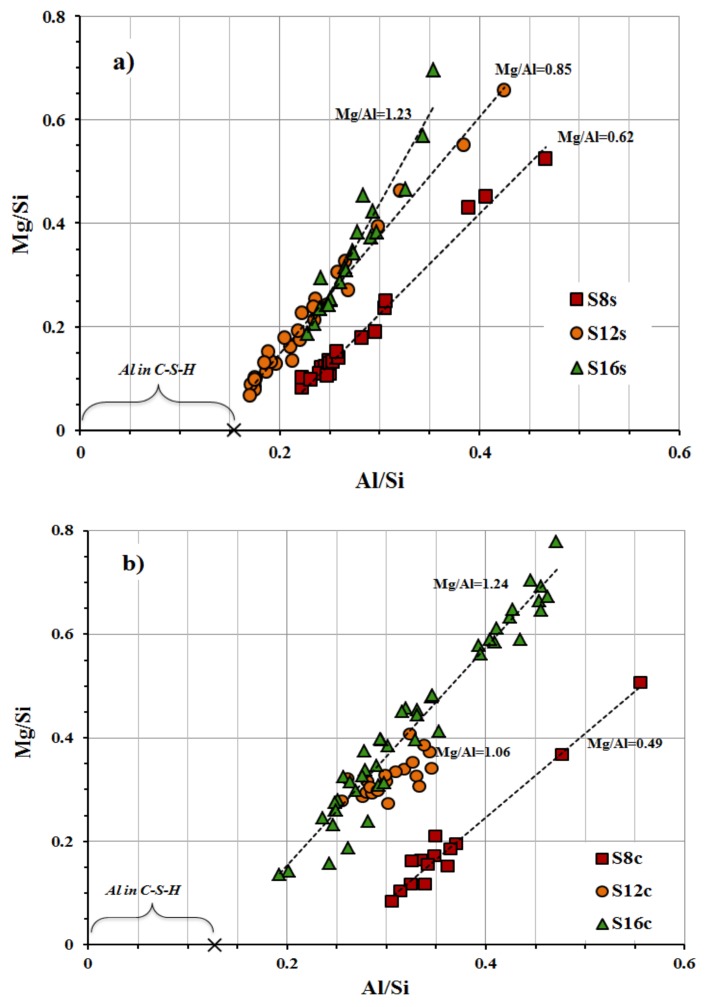
EDX analysis results after 28-day show the effect of the atomic ratio Al/Si versus Mg/Si of the different slags activated with (**a**) 10% sodium silicate SS and (**b**) 10% sodium carbonate SC.

The carbonation of the studied mixes was determined on 3 months old specimens stored in ambient laboratory conditions at 20 ± 2 °C and 50 ± 10% RH. The highest carbonation developed in SC activated mixes based on the slag S_12_, Figure 9. While, the SS activated mortars based on the slag S**_8_** showed the least extensive carbonation. The high carbonation resistance can be related to a higher SiO_2_, Al_2_O_3_ content and fineness, which led to faster reactions resulting in denser microstructures and higher amounts of formed C-(A)-S-H phases. Comparing mixes based on coarser slags S**_12_** and S**_16_**, the results indicated that an increasing MgO content improved the carbonation resistance which is in agreement with earlier studies [14]. The higher carbonation resistance of the S**_8_** mix having the lowest MgO content can be related to its higher fineness. The MgO content effected the chemical composition of the formed phases. The calculated Al/Si vs Mg/Si ratios varied between 0.49 and 1.24 for SC-activated mixes and 0.62 and 1.23 for mixes activated with SS, Figure 10. According to earlier studies, those results indicate the formation of hydrotalcite and as well as less incorporation of Al into the C-S-H phase [20]. The incorporation of Al in the C-S-H decreased with an increased MgO content and resulted in a lower 28-day compressive strength indicating better mechanical properties of the C-A-S-H phase in comparison with hydrotalcite or gaylussite, Figure 10.

The EDX analysis indicated that all mixes contained C-S-H phases. The Ca/Si ratio was between 1.0 and 1.55 for all mixes except for the mix S**_12c_** that had the Ca/Si ratio of 3.2, Table 2). The very high Ca/Si ratio of the mix S**_12c_** indicated the presence of silicate chain units Q^1^ and portlandite, which could be related to its high carbonation degree [24]. The XRD analysis did not detect hydrotalcite despite indications from earlier experimental results and thermodynamic modelling.

The EDX analysis results showed the formation of hydrotalcite which increased by increasing the MgO content and decreased at lower Na_2_O% concentrations [17,25,26,35].

**Figure 11:**
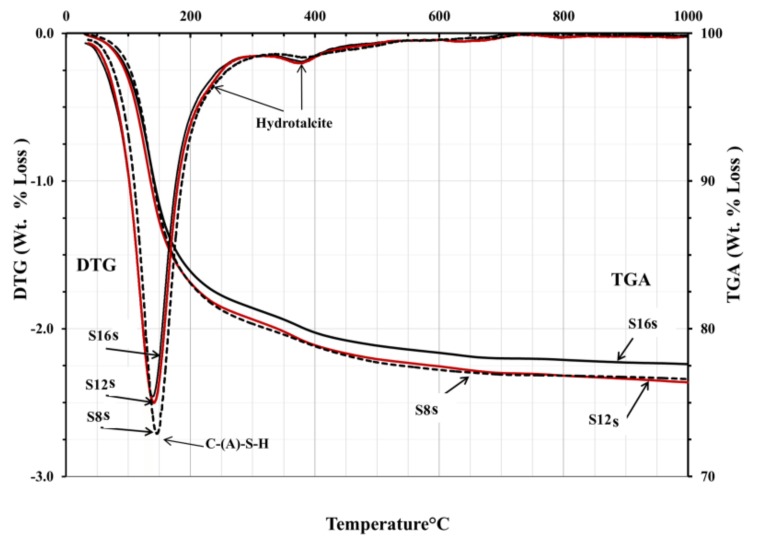
DTG and TGA results of AASs pastes activated with sodium silicate SS at age 28-day.

**Figure 12:**
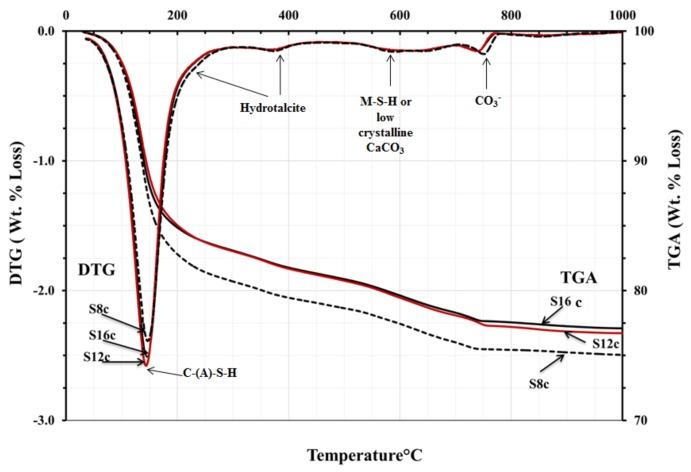
DTG and TGA results of AASs pastes activated with sodium carbonate SC at age 28-day.

The TGA/DTG analysis showed the decomposition peaks between 200 and 400 °C, which could be identified as hydrotalcite-like phases, Figure 11 and Figure 12. Consequently, neither presence nor absence of hydrotalcite could be confirmed based on the obtained tests results. However, based on earlier results and taking into account the high MgO content, such trend should be considered as very likely. The TGA test results were very similar for all mixes. All mixes showed peaks related directly to the decomposition of C-S-H related phases located between 100 and 200 °C. An exception were mixes activated with SC carbonate where calcite or magnesium silicate hydrate M-S-H were identified as possible phases, Figure 12. The peak observed at 500–600 °C was presumably related either due the dehydration of thomsonite (NaCa_2_Al_5_Si_5_O_20_·6H_2_O) [37], M-S-H gel [21], or the decomposition of a poorly crystallised CaCO_3_ [38].

Other oxides including especially CaO and Al_2_O_3_ were shown by others to affect the mechanical properties of alkali-activated binders. For example decreasing CaO and Al_2_O_3_ contents of slag caused a strength reduction [39]. The present results complied with this finding in the case of the CaO but were contradicting in the case of Al_2_O_3_, which could be related to the significantly finer particles size distribution of the S**_8_** slag.

**Figure 13:**
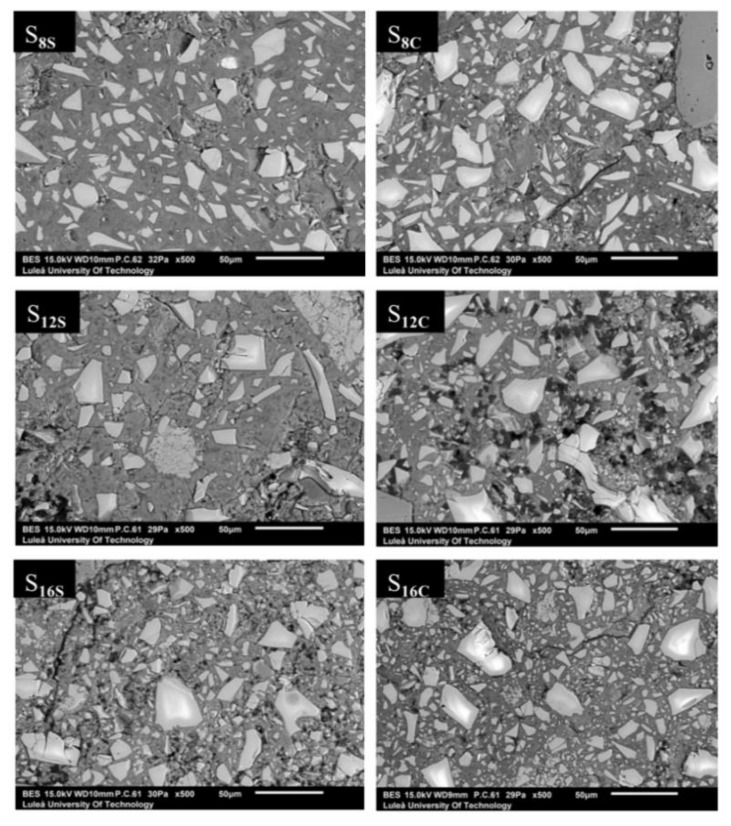
SEM images of the AASs mortars after 28 days of sealed curing at 21 ± 2 °C and 35 ± 5% RH.

The SEM/BSE analysis of 28-day mortars showed a significant variation in porosity, Figure 13. The lowest porosity and the most homogenous binder matrix was observed in mortars made of S**_8_** slag which complies with the highest develop strength results. The other two slags S**_12_** and S**_16_** being coarser and with higher MgO content, their microstructure appeared visibly more porous. The extreme case was the mix S**_12_** activated with SC, which also had the lowest 7-day compressive strength.

**Figure 14:**
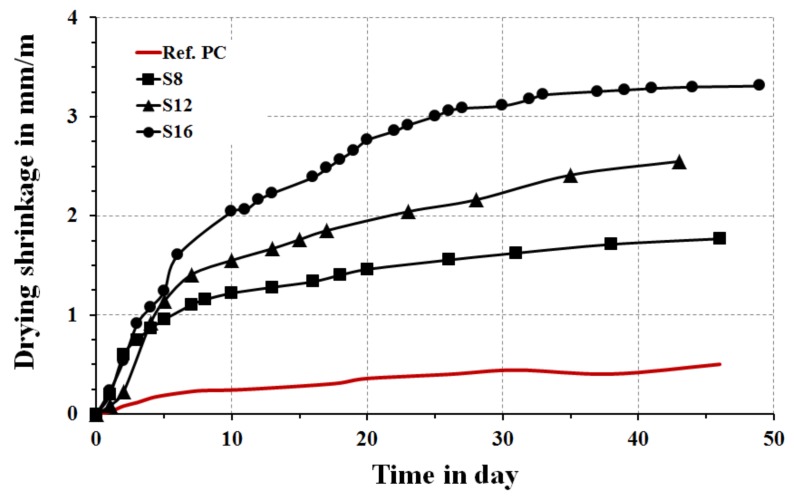
Drying shrinkage results of AASs concretes with reference PC concrete mix.

The shrinkage values were measured for concrete samples activated with 10 wt % SS to verify the effects of the slag, Figure 14. As expected, shrinkage values were higher in comparison with the reference concrete base on the Portland cement. The lowest values in between the alkali-activated mortars were measured for the S**_8_** mix, which could be attributed to the fastest reaction rates, which led to form a stronger binder structure having higher resistance to shrinkage. Higher MgO content increased the drying shrinkage.

**Figure 15:**
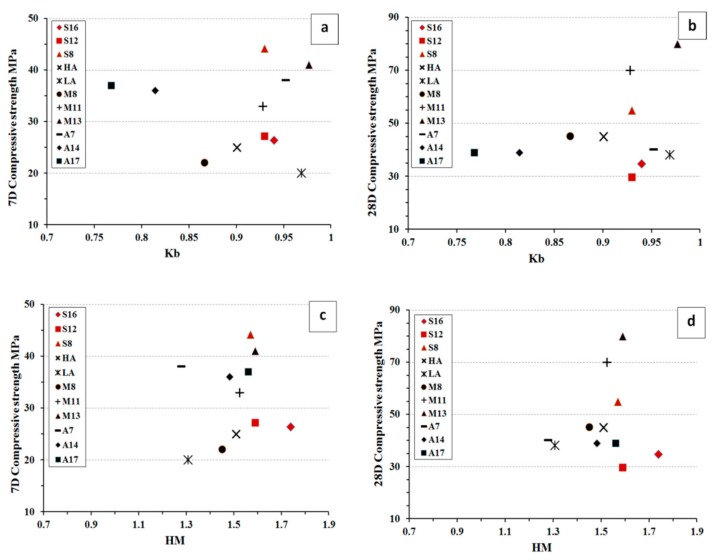
Dependencies between (**a**,**b**) Kb with 7-day and 28-day compressive strength respectively, (**c**,**d**) HM with 7-day, 28-day compressive strength respectively. Comparative results were adapted from others [23,30,40,41].

The comparative analysis of basicity modulus (K_b_) and the hydration modulus (HM) versus the 7 and 28 d compressive strength are shown in, Figure 15. The Kb value was calculated as K_b_ = (CaO + MgO)/(SiO_2_ + Al_2_O_3_) and the HM as HM = (CaO + MgO + Al_2_O_3_)/SiO_2_). The data included values for the three types of slag used in this study and 8 other slags used by others [23,30,40,41]. All slags were activated with liquid sodium silicate SS and had fineness values showed in Table 3. The analysis showed that the fines has a stronger correlation on the developed strength values that the calculated Kb and HM values. There was not significant correlation find between Kb and HM values and the 7 or 28 d compressive strength values. The situation was the same for the studied in this research slags and the data adopted from others.

## 4. Conclusions

Selected properties of concretes based on three different GGBFS alkali-activated with 10 wt % sodium silicate SS and sodium carbonate SC were investigated. The used slags varied only slightly in the chemical compositions but had different MgO contents and variable fineness. In general, the performed experimental study showed that the specific surface area of slags had a greater impact on the microstructure, mechanical properties, shrinkage, efflorescence, and carbonation than their chemical composition. The GGBFS activated with SC had in general longer initial and final setting times in comparison with the SS-activated slags. Mixes based on the GGBFS having the coarsest particles size distribution and the lowest Al_2_O_3_ content showed the longest initial and final setting times. GGBFS with the fineness particles, higher content of SiO_2_, CaO and Al_2_O_3_ developed the highest hydration temperatures, the lowest flowability and the highest early and ultimate compressive strengths independently of the used alkali activator. However, those mixes showed also the most extensive micro-cracking when activated with the SC. The most intensive efflorescence was observed in SS-activated mixes containing coarser slag. In general, the SS-activated mixes showed a lower carbonation than the SC-activated, while the increased fineness enhanced the carbonation resistance regardless of the MgO content and decreased the shrinkage of AAS concrete. The increased MgO content decreased the Al uptake by the C-S-H. Activation with SC resulted in a predominant formation of the C-(A)-S-H phase accompanied by secondary formation of hydrotalcite, calcite, and M-S-H. Mixes activated with SS showed mainly the formation of C-(A)-S-H and hydrotalcite-like phases. An increasing MgO content increased the early age drying shrinkage. 

## Figures and Tables

**Table 1:** The chemical compositions and physical properties of the slags.

Oxides	S_16_	S_12_	S_8_
SiO_2_	35	34	37.9
Al_2_O_3_	14.3	11.6	13.2
CaO	30.4	30.3	38.5
Fe_2_O_3_	0.3	0.3	0.37
K_2_O	0.7	0.8	0.6
MgO	16.1	12.1	7.8
MnO	0.5	0.5	0.2
Na_2_O	0.6	0.5	0.5
P_2_O_5_	<0.01	<0.01	<0.01
TiO_2_	2.8	2.1	0.8
LOI	−0.9	−0.9	−1
MgO/Al_2_O_3_	0.88	1.04	0.6
Ca/Si	1.33	1.36	1.55
Mg/Al	1.28	1.18	0.67
Kb	0.94	0.93	0.91
HM	1.74	1.59	1.57
Specific surface area m^2^/kg	450	435	498
Compacted Density gm/cm^3^	2.95	2.95	2.90

**Table 2:** Mortars and pastes mix proportion.

Mix ID	w/b Ratio	Slag gm	Sand B35 gm	Activator Type	Activator Dose as Solid Content	Ca/Si Ratio in AAS Pastes after 28 d of Sealed Curing
Mortars S_16S_	0.45	35	35	SS	10 wt %	
S_12S_	0.45	35	35	SS	10 wt %	
S_8S_	0.45	35	35	SS	10 wt %	
S_16C_	0.45	35	35	SC	10 wt %	
S_12C_	0.45	35	35	SC	10 wt %	
S_8C_	0.45	35	35	SC	10 wt %	
Pastes S_16S_	0.36	35	---	SS	10 wt %	1.02
S_12S_	0.36	35	---	SS	10 wt.%	1.15
S_8S_	0.36	35	---	SS	10 wt %	1.25
S_16C_	0.36	35	---	SC	10 wt %	1.17
S_12C_	0.36	35	---	SC	10 wt %	3.22
S_8C_	0.36	35	---	SC	10 wt %	1.55

**Table 3:** The fineness values of the slag used for comparison in Figure 15. S_16_, S_12_, and S_8_ are slags used in the present study and remaining slags were used by others [23,29,38,39].

Slag	S_16_	S_12_	S_8_	HA	LA	M8	M11	M13	A7	A14	A17
Fineness m^2^/Kg	450	435	498	502	503	499	507	501	502	496	499
